# On Syncope Angens

**Published:** 1817-02

**Authors:** James Johnson


### *On* SYNCOPE ANGENS.

By JAMES JOHNSON, M. D.

ALTHOUGH, since the ingenious and excellent publication of Dr. Parry on Syncope Angens, in 1799, the disease has excited much attention, and its pathology has been considered as nearly settled, yet many physicians, and some writers of eminence, do not view it in the same light as Dr. Parry has done. Dr. Parr, in his London Medical Dictionary, states it as his opinion, that, "the cause is most probably a *spasm* or convulsion;" and denies that ossification of the coronary arteries is any thing but a concomitant symptom, or rather an effect of angina pectoris. M. Valerien Louis Brera, Clinical Professor in the University of Padua, has also published a new theory of the disease, denominating it "*sterno-cardia*," and does not consider it as arising from any organic derangement of the heart itself, but from an oppressed state or impeded function of that organ. Dr. Parr, however, does not bring forward any facts in support of his position; and he commits a mistake in saying of Dr. Parry's patients, that "in the *only* case where ossifications were found, the disease was obscurely marked; while the others, more certainly cases of angina pectoris, offered no such appearance." From this sentence one would conclude that Dr. Parr had never read the work he was criticising. The case of Mr. Carter, at Dursely, exhibited coronary ossification, (page 3 of Dr. Parry's work). The case of Mr. Bellamy, related by Mr. Paytherus, did the same; or at least, shewed cartilaginous obstruction in the nutrient vessels of the heart (page 12). The Reverend Mr. S. under Dr. Parry's inspection, shewed "the two coronary arteries ossified" (page 24). Mr. M's coronary arteries "contained within their cavities hard incrustations, which could easily be drawn out, and appeared to be bony tubes," page 33. Dr. Parr's testimony can therefore weigh nothing in the scale.

In respect to the Italian Professor's theory, if we may be permitted to judge by some cases of his, translated into the fifth volume of the *New Medical and Physical Journal*, we should not expect much accuracy of discrimination from that quarter. The first case exhibited no symptom of angina pectoris, except pain in the left arm, which we know to accompany many other organic lesions of the heart, and to be frequently wanting in true angina pectoris. The manner of the poor man's death too, was unlike that from syncope angens. "He was seized with a violent pain in the head, and fell down senseless," where he instantly died. The liver was enlarged and hard, and its left lobe was raised in such a manner as to forcibly push up the posterior inferior surface of the heart, and retain that viscus in a state of total compression. The right side of the heart itself was morbidly dilated.

The following is another specimen of our author's angina pectoris:—"A man, ætat. 40, of an athletic make, and rather corpulent, much addicted to wine and spirits, was suddenly seized in the night with a sense of suffocation, and instantly expired."—All the previous symptoms that M. Brera learned, were "convulsive attacks in the chest;" to which this man had been long subject! The liver here was large, and encroached on the lungs and heart. The *right* side of the latter, as might have been expected, was passively dilated.

From these and other proofs of total want of discrimination on the part of the Italian Professor, we may pass over his theory and nosological designation of syncope angens, without farther comment. They only tend to show, in a still clearer light, the superior accuracy and judgement of our own countryman.

The case of Dr Black, of Newry, in the fourth volume of the Memoirs of the Medical Society, strikingly corroborates Dr. Parry's assumptions,* as does the experience of Dr. Jenner and Allan Burns. As it rarely happens that the medical attendant has the opportunity of seeing the phænomena of the fleeting paroxysms of this terrible disease; and as the history and dissection of the following case contain some peculiar traits, which may throw some additional light on the *ratio symptomatum,* I trust they will prove interesting.

On the 9th of July, 1816, I was called in great haste, at seven in the evening, to Peter Myrtle, a messenger, in the Dock-yard at this Port, aged 40 years, whom I found apparently dying. He was gasping for breath, yet endeavouring to retain deep inspirations; his right arm was raised over his head, and clinging by a door, while his wife was rubbing the thorax, about the region of the heart, and another woman was chafing the left arm, which appeared pale and lifeless. His face was pallid, but expressive of exquisite distress; the lips were livid. The forehead bathed in a cold perspiration. The pulse accelerated, rather weak, but not sensibly irregular or intermitting. He could scarcely articulate, in consequence of the pain in the region of the heart and in the left arm, and also of the sense of suffocation.

Without waiting to learn the history of the case, as the

---

* Dr. Black, of Newry, has lately communicated in the seventh vol. of the Medico-Chirurgical Transactions, two cases of angina pectoris, in addition to those formerly stated. Ossification of the coronary arteries was found in both, with large flaccid heart: but hydrothorax appears to have been the immediate cause of death in these subjects. The dissections are not very accurately stated, nor the attendant symptoms clearly or minutely detailed; but the general results confirm Dr. Parry's pathology of the disease.

man appeared to have but a few minutes to live, if not relieved, I conceived that circumstances authorised immediate venæsection, and I instantly opened a vein. The blood was jet black, and till about sixteen ounces were in the bason, no particular relief was obtained. From this time until twenty-four ounces were abstracted, the pain in the cardiac region gradually subsided; and when the bandage was applied to the orifice, he sat down and calmly related the following outline of his case.

About five years ago, he fell down a trap-hatch in the rigging loft, and pitched with his left breast against the opposite combing, right on the region of the heart. He was taken up apparently lifeless; but he soon recovered, and although he felt pain for a few days, he never afterwards thought more of the accident.

In April, 1815, while walking in the street, he was suddenly seized with a violent pain about the guard of the left arm, which pain presently shot into his breast, and was accompanied by a loss of breath, and a sensation as though he were dying. With much difficulty he reached a neighbouring public-house, and immediately swallowed a glass of gin, (the remedy for all diseases) and soon afterwards found himself much relieved.

Since that period, he has never passed a day entirely free from a paroxysm or paroxysms of this kind, more or less severe. They generally come on about five o'clock in the afternoon, while he is walking home from the dockyard; but they are readily excited at any time, by an unusual degree of corporeal exertion or mental agitation. He is frequently seized with a paroxysm in the night, especially if he wakes suddenly from a frightful dream, to which he is occasionally subject. For a long time past, the pain in the region of the heart is accompanied by a most distressing palpitation, and sensation, as though the organ of circulation were rolling over and over. He also asserts, that he feels the heart sometimes cease, as it were, to beat. The paroxysm seldom lasts longer than ten or fifteen minutes, but sometimes an hour or more. There is always, even between the paroxysms, an habitual dyspnœa.

The last paroxysm, before described, was by far the severest he ever experienced, and he is convinced that he could not have survived ten minutes after I arrived, had he not found relief. Latterly, he had been occasionally troubled with hæmorrhoids; and while they protruded externally, and especially when they bled, the palpitation and other symptoms were proportionally milder. During the last three or four paroxysms, he was much distressed with a severe globus hystericus, in addition to the other symptoms. At all times flatulence and borborygmi were frequent and troublesome, and eructations constantly gave relief.

The case now appeared to me to be one of syncope angens, and the large bleeding which I had ventured on before investigating the case, was looked back on with a temporary alarm at my rashness; the remedy being rather heterodox, according to the best writers on the subject. But this alarm soon ceased, on reflecting on the immediate benefit which had evidently resulted from the operation. A draught, with carb. ammon. and æther; at night, two cathartic pills.

He had been under several medical practitioners, all of whom, I believe, considered the disease as arising from some organic lesion of the heart, the exact nature of which they could not develope. He had experienced some alleviation of his sufferings from abstinence, open bowels, and digitalis. He had also been once bled, but not during a paroxysm.

*July* 10th. He has passed an easy and quiet night; but the bowels are confined. Another brisk cathartic. In the presence of Dr. Denmark, I this day examined my patient, in the recumbent and other positions, with all possible care and minuteness. Abdominal pressure gave little uneasiness, till it was carried so far as to cause much encroachment on the capacity of the chest, when pain, or rather a sense of oppression, was complained of about the heart. A slight appearance of pulsation was visible at the epigastrium, but could scarcely be felt. The descending aorta could be distinctly traced pulsating to its bifurcation in the loins. Thoracic percussion led to nothing. The beat of the heart against the ribs was not very distinct, however; in short, there was nothing gathered from this examination that decisively indicated what the cardiac lesion was, and whether functional or structural.

This poor man was perfectly aware of the danger, and in some measure of the *nature* of his complaint; since he said he was certain there was "*something growing to his heart.*" He assured Dr. Denmark and myself (for he attended minutely to every sensation, and kept a journal of his disease), that the pulse in the left wrist sometimes beat but half the number of pulsations of that in the right. The pain and numbness of the left arm, at first was in the middle, then descended to the elbow, and for some time past came down to the points of the ring and little fingers, but never affected the others. The direction of this pain was not in the line either of the nerves or arteries.

Viewing the disease as *organic* lesion of the heart, or in all probability of obstruction in the coronary vessels, it was evident that the *function* of the organ was only occasionally disturbed, and consequently remedial measures could only be directed against the exciting causes of these paroxysms. Among these, vascular plethora, vascular action produced by muscular exertion, and irritability, appeared the most important. Abstinence, repose, open bowels, and sedatives, were therefore prescribed. Hyoscyamus was combined with calomel and extract of colocynth; to which was added the tartrite of antimony, to act on the stomach and extreme vessels of the skin. The flatulence, which was much complained of, was obviated by ammonia and bitters.

For two days after the bleeding, he had no return of the pain or dyspnœa, except a slight sense of constriction while straining at stool. Such an immunity he had not experienced for many months before. He now informs me, that he is habitually costive, generally two, three, or four days without a stool; and what is singular, he assures me, that, when constipated, he always feels better than when relaxed. A paroxysm is not unusual after a lax motion.

*July* 13th. He this day gave me his journal, in which he has noted, with the most scrupulous exactness, every pain he has felt during the last fifteen months, with the hours of their occurrence, and every other circumstance. It appears from this, that, during the last two months, he has had from three to six paroxysms every day; but principally between four in the afternoon and twelve at night. Various have been the concomitant morbid sensations: the left arm was almost always affected, never the right. Often the kidnies were the seat of pain; sometimes other parts of the body. He has had slight and transient paroxysms within the last twenty-four hours. He continues his medicines; with a blister to the sternum.

*July* 14th. Has had two or three severe, and numerous slight paroxysms since last report. Last night, however, the pain lasted all night, so that he could not sleep, nor lie down at all. He had taken some opening medicine yesterday, which operated three times. I was sent for this morning. He was down stairs, but still in pain. The pulse in the left arm was synchronous with that in the right, but feeble. Neither radials were synchronous with the action of the heart. Gave him tinct. valer. ammon. ʒj, sp. æth. sulph. ʒß, aq. menth. v. ℥jß, statim sumend. He felt worse for a few minutes, but was shortly seized with an inclination to make water, and voided a pint, after which he felt relieved. Ordered him the following medicine:

℞. Infusi valerianæ ℥vj, carbonatis ammoniæ ʒß, tinct. assafœt. ʒj, tinct. opii ʒj. M. capiat coch. ij. mag. pro re nata quando dolor adsit.—℞. Extracti hyosciami, pilulæ hydrarg. āā ℈j, ex. opii, g. v. ft. pilulæ xv. Capiat ij omni nocte.

He says the pain is more towards the epigastrium than formerly; feels pain in the kidnies.

*July* 27th. He had some transient pains in the region of the heart yesterday, but towards evening he was pretty easy, went to bed, and slept some hours. At one this morning he awoke in great agonies, and said he was dying. He sent for me to bleed him, but I was unable to go. One of my pupils visited him, and found him in a paroxysm similar to that in which I first saw him. He abstracted a few ounces of blood; but although his breathing became somewhat easier, the painful sensations about the heart continued, and at three o'clock he expired.

*Sectio Cadaveris, 28th July,* 1816. *Present Dr. Lara, Dr. Denmark, and two of my Pupils.*

A thick layer of intensely yellow fat on the abdomen and thorax. Lungs on the right side adherent to all parts of the pleura costalis, diaphragm, and pericardium; but healthy in structure. On the left side there were only partial adhesions. The pericardium being slit open, was found to contain about an ounce of bloody serum. The heart itself was large and fat, curiously mottled on its surface, and remarkably soft and flabby. The right auricle, which was first opened, was empty; its parietes were extremely thin, pale, and flaccid. The musculi pectinati auriculæ dextræ very pale, flabby, and slender. The auriculo-ventricular opening rather large; no apparent disease or imperfection in the tricuspid valves. The right ventricle, which was also empty, was considerably enlarged passively, its parietes being in the same thin and weak state as those of the corresponding auricle. No ossification in any part of this side of the heart. The pulmonary artery and sigmoid valves natural. The left auricle, which was full of black fluid blood, presented nothing unnatural, except the paleness, softness, and weakness of its muscular bands and parietes, and its passive dilatation. The left ventricle was in the same state, and full of black blood. Where the cordæ tendineæ are inserted into the margins of the valvulæ mitrales, incipient points of ossification and cartilage were found. The origin and arch of the aorta were very large, and the coats thickened; it was full of black fluid blood. The root of the aorta, for about half an inch, viz. that ring of it which is occupied by the semilunar valves, was considerably thickened, indurated, and containing several bony scales, and still broader patches of cartilage. The valves themselves were not diseased. The right coronary artery was seen opening into the aorta quite clear of the semilunar valves. A probe was passed into it, and the vessel was slit open from its origin to the apex of the heart. Not a speck of disease or ossification was found. The origin of the left coronary artery was long sought for, but in vain. I therefore made a transverse section of the heart, when the left coronary artery was immediately recognized. A probe was then passed into it, towards the aorta. The probe went on freely till its point came to the thickened ring of the aorta, but would proceed no farther. It would not enter the aorta, till forcibly pushed through. That part of the coronary artery, between its origin and the transverse section of the heart, was next slit open, but exhibited no disease. The probe was then passed through the other division of the vessel to its termination, and the vessel laid open, but no disease appeared; the vessel however was small. Abdominal viscera natural, except that the duodenum was larger than the transverse arch of the colon, and it appeared like one half of the stomach, the pylorus resembling a central stricture. The stomach itself was distended, to the utmost possible degree, with wind; as were all the intestines, in which no fæcal remains were to be found.

*(Some Observations on this Case will be inserted in the following Number.)*

*Oriental*

